# Association between menstrual cycle irregularity and tinnitus: a nationwide population-based study

**DOI:** 10.1038/s41598-019-50559-5

**Published:** 2019-10-01

**Authors:** Jin-Na Yu, Ga Eun Nam, Kyungdo Han, Ji-su Kim, Yang-Hyun Kim, Kyung Hwan Cho, Gunseog Kang, Yong Kyun Roh

**Affiliations:** 1 Department of Nursing, Chung-Ang University, Seoul, 06974 Republic of Korea; 2 Department of Family Medicine, Korea University Anam Hospital, Korea University College of Medicine, Seoul, 02841 Republic of Korea; 3 Department of Biostatistics, College of Medicine, The Catholic University of Korea, Seoul, 06591 Republic of Korea; 4 Department of Statistics and Actuarial Science, Soongsil University, Seoul, 06978 Republic of Korea; 5 Department of Family Medicine, Kangnam Sacred Heart Hospital, Hallym University College of Medicine, Seoul, 07441 Republic of Korea

**Keywords:** Epidemiology, Reproductive signs and symptoms

## Abstract

This population-based cross-sectional study investigated the association between menstrual cycle irregularity and tinnitus in premenopausal Korean women. We used data from the 5th Korea National Health and Nutrition Examination Survey (2010–2012). A total of 4633 premenopausal women were included. Hierarchical multivariable logistic regression analysis was performed. Individuals with tinnitus accounted for 21.6%. Women with tinnitus or menstrual irregularity had significantly higher rates of stress, depressive mood, and suicidal ideation than those without. The proportion of individuals with irregular menstrual cycles with duration of longer than 3 months increased as the severity of tinnitus increased (P = 0.01). After adjusting for confounding variables, the odds of tinnitus increased in individuals with irregular menstrual cycles compared to those with regular menstrual cycles. The odds ratios (ORs) of tinnitus tended to increase as the duration of menstrual irregularity became longer (1.37, 95% confidence interval: 1.06–1.78 for duration of up to 3 months; 1.71, 1.03–2.85 for duration of longer than 3 months, P for trend = 0.002). Our study found a positive association between menstrual cycle irregularity and tinnitus. Menstrual cycle irregularity may be a related factor of tinnitus in women with childbearing age.

## Introduction

Tinnitus refers to a perception of sound even in the absence of external auditory stimuli^1^. The prevalence of tinnitus has been reported to be between 5.1% and 42.7% worldwide^1^. In Korea, the prevalence is reported to be 20.7%, and the proportion of those who experience annoyance due to tinnitus is as high as 30%^2^. The prevalence of tinnitus and annoyance caused by tinnitus increases with age^2^, and the annoyance seems to be more severe with a longer durations^3^. People with tinnitus may also have health problems such as hearing loss, sleep disturbance, and hyperacusis^4^. However, this highly debilitating condition is difficult to cure and there are no satisfactory treatments^5^. Therefore, it is very important to evaluate and control modifiable risk factors of tinnitus.

People with tinnitus are more likely to complain of mental health problems. Lifetime prevalence of anxiety disorders reaches 45% among individuals with tinnitus^6^. They experience lower self-esteem and well-being^7^, and moreover, 7.2% of patients with severe tinnitus had ever attempted suicide^8^. Tinnitus was also associated with stress^9^ and hypothalamic-pituitary-adrenal (HPA) axis dysregulation, which is found in stress-related disorders^10^. Abnormally increased levels of stress related hormones such as norepinephrine or 5-hydroxyindoleacetic acid were observed in people with tinnitus^11^ and tinnitus may be exacerbated by stress and lack of sleep^12^. In particular, psychological symptoms observed in patients with tinnitus aggravate the distress experience due to tinnitus, and subsequently decrease their quality of life^13^. The odds of tinnitus and annoying tinnitus were reported to be higher in women than men^14^. Also, women experience a greater degree of tinnitus-related distress than men^15^. Moreover, various mental health characteristics in women with tinnitus may be related to their menstrual cycle^16,17^. Therefore, it appears that tinnitus needs to be managed carefully, especially in women.

Menstrual cycle regularity is a non-invasive clinical parameter to assess female physical health status or reproductive capacity, and is influenced by age, lifestyle, and various physical conditions^18–20^. Menstrual cycle irregularities are common in women of childbearing age^21,22^, and have been reported to be associated with various chronic diseases such as diabetes, cardiovascular disease^23^, and breast cancer^24^ as well as infertility^25^. Menstrual cycle irregularity is also associated with mental health characteristics in women^17,26^. Psychological factors such as anxiety and depression are risk factors for polycystic ovary syndrome (PCOS), which is known to cause menstrual cycle irregularity^27^. In this way, both tinnitus and menstrual cycle irregularity seem to be closely related to the physical and mental health in women. In addition, a previous study reported higher prevalence of tinnitus in pregnant than in non-pregnant women and suggested a relationship between female hormonal changes and tinnitus^28^. However, to the best of our knowledge, the relationship between menstrual cycle irregularity and tinnitus has been scarcely studied.

Therefore, we investigated the association between menstrual cycle irregularity and tinnitus in women of childbearing age by using nationwide representative data from the South Korean population.

## Results

### Characteristics of study participants according to the presence of tinnitus

Table 1 shows the characteristics of study participants according to the presence of tinnitus. Of the total participants, 21.6% reported experiencing tinnitus. The mean age was 35.8 ± 0.2 and 34.0 ± 0.4 years in individuals without and with tinnitus, respectively (P < 0.001). The proportion of menstrual cycle irregularity was higher in participants with tinnitus than those without (P = 0.001). The group with tinnitus showed lower education and income levels than the group without tinnitus (P = 0.007 and <0.001, respectively). There were no differences in residential areas and occupations between the two groups. The participants with tinnitus had a higher rate of smoking than those without tinnitus (P = 0.003), however, alcohol drinking, physical activity, and sleep duration did not differ between two groups according to tinnitus. The women without tinnitus had significantly more experience of childbirth than those with tinnitus, while rate of oral contraceptive use were similar between two groups. BMI was slightly higher in individuals without tinnitus than those with tinnitus (P < 0.001).

**Table 1:** Characteristics of study participants according to the presence of tinnitus.

N	Tinnitus		*p*-value*
	No	Yes	
	3699	934	
Age (years)	35.8 ± 0.2	34.0 ± 0.4	<0.001
Menstrual cycle irregularity	14.1 (0.7)	19.7 (1.7)	0.001
Educational level			0.007
≤Elementary school	2.7 (0.3)	4.7 (0.8)	
Middle school	5.7 (0.5)	5.8 (0.8)	
High school	48.0 (1.1)	51.3 (2.0)	
≥University	43.6 (1.1)	38.2 (2.0)	
Household income level			<0.001
Q1	7.7 (0.7)	12.7 (1.5)	
Q2	28.0 (1.1)	28.6 (1.9)	
Q3	32.7 (1.0)	29.5 (1.7)	
Q4	31.6 (1.1)	29.2 (1.9)	
Residential area (urban)	85.8 (1.5)	87.5 (1.8)	0.282
Occupation (unemployed)	42.1 (1)	44.6 (2.1)	0.273
Ex-smokers or current smokers	9.1 (0.6)	13 (1.4)	0.003
Heavy alcohol drinkers	3.3 (0.4)	2.9 (0.7)	0.590
Regular exercisers	83.3 (0.8)	82 (1.7)	0.452
Sleep duration (hours)	7.0 ± 0.0	7.0 ± 0.1	0.288
Parity (yes)	67.7 (1.1)	58.3 (2.1)	<0.001
Ever use of oral contraceptives (yes)	8.9 (0.6)	9.9 (1.2)	0.397
Body mass index (kg/m^2^)	22.6 ± 0.1	22.5 ± 0.2	<0.001

Data are presented as mean ± standard error or percentage (standard error).

*p-values were obtained using an independent t-test for continuous variables and a chi-square test for categorical variables.

### Mental health characteristics according to the presence of menstrual cycle irregularity and tinnitus

Table 2 shows mental health characteristics according to menstrual cycle irregularity and tinnitus. Individuals with menstrual cycle irregularity had higher rates of experiencing stress (P = 0.001), depressive mood (P < 0.001), and suicidal ideation (P < 0.001) than those with regular menstrual cycle. Similarly, women with tinnitus also had higher rate of experiencing all these mental health problems than those without tinnitus (P < 0.001).

**Table 2:** Mental health characteristics according to the presence of menstrual cycle irregularity and tinnitus.

	Stress (yes) (n = 1494)	Depressive mood (yes) (n = 640)	Suicidal ideation (yes) (n = 673)
Menstrual cycle irregularity			
No	32.4 (0.9)	13.5 (0.7)	14.9 (0.7)
Yes	40.0 (2.2)	20.5 (1.7)	21.8 (1.9)
*p*-value*	0.001	<0.001	<0.001
Tinnitus			
No	31.3 (1.0)	13.0 (0.6)	13.7 (0.7)
Yes	41.8 (2.0)	19.9 (1.6)	24.2 (1.8)
*p*-value*	<0.001	<0.001	<0.001

*p-values were obtained using a chi-square test.

Data are presented as percentage (standard error).

### Distribution of menstrual cycle characteristics according to the tinnitus severity

**Figure 1:**
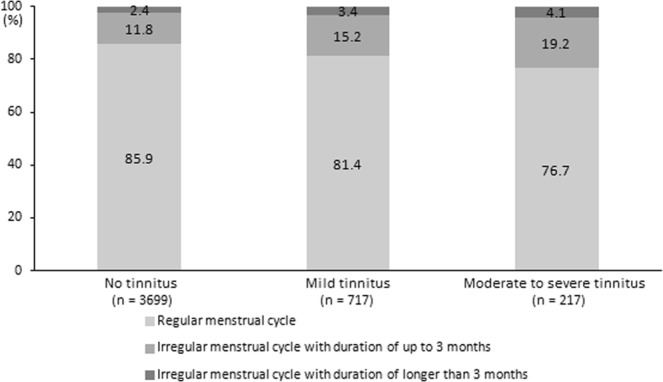
The distribution of menstrual cycle characteristics according to tinnitus severity (P = 0.01 by using a chi-square test).

Figure 1 shows the proportion of menstrual cycle characteristics according to tinnitus severity. As the severity of tinnitus increased, the proportion of individuals with a regular menstrual cycle decreased. Also, the proportion of those with irregular menstrual cycles with duration of longer than 3 months increased with more severe tinnitus (P = 0.01).

### Odds of tinnitus according to menstrual cycle characteristics

Table 3 shows the adjusted ORs (95% CIs) of tinnitus according to menstrual cycle characteristics. After adjustment for age, smoking status, alcohol intake, physical activity, parity, and BMI (model 1), the ORs of tinnitus were greater in the irregular menstrual cycle group than in the regular menstrual cycle group (1.41, 95% CI: 1.09–1.82 for irregular menstrual cycle with duration of up to 3 months; 1.77, 95% CI: 1.05–2.97 for irregular menstrual cycle with duration of longer than 3 months, P for trend = 0.001). These associations between menstrual cycle irregularity and tinnitus persisted even after further adjustment for educational level, household income level, sleep duration, use of oral contraceptives, psychological stress, and depressive mood (model 2). The ORs of tinnitus were 1.37 (95% CI: 1.06–1.78) for women with irregular menstrual cycles with durations of up to 3 months and 1.71 (95% CI: 1.03–2.85) for those with irregular menstrual cycle with durations of longer than 3 months (P for trend = 0.002).

**Table 3:** Odds ratios (95% confidence intervals) of tinnitus according to menstrual cycle characteristics.

	Model 1	Model 2
Regular menstrual cycle	1	1
Irregular menstrual cycle with duration of up to 3 months	1.41 (1.09–1.82)	1.37 (1.06–1.78)
Irregular menstrual cycle with duration of longer than 3 months	1.77 (1.05–2.97)	1.71 (1.03–2.85)
*p* for trend	0.001	0.002

Model 1 was adjusted for age, smoking status, alcohol intake, physical activity, parity, and body mass index.

Model 2 was adjusted for age, smoking status, alcohol intake, physical activity, parity, body mass index, educational level, household income level, sleep duration, use of oral contraceptives, psychological stress, and depressive mood.

## Discussion

This study was conducted to investigate the association between menstrual cycle irregularities and tinnitus among premenopausal women. There was a significant positive association between irregular menstrual cycles and the odds of tinnitus. Especially, odds of tinnitus increased as the durations between the menstrual cycles were longer. These associations between menstrual cycle irregularity and tinnitus were continued irrespective of adjustment for potential confounding variables regarding sociodemographic, lifestyle, physical, and mental health characteristics. Our findings suggest that menstrual cycle irregularity may be a risk factor of tinnitus in premenopausal women.

Previous studies reported that tinnitus patients often experience mental health problems such as stress^12^, and anxiety^6^. Also, mental health status has been reported to affect regularity of menstrual cycles^16,17,27^. However, no study has explored the relationship between menstrual cycle irregularity and tinnitus. Interestingly, a few studies have shown that hormonal changes during menstrual cycles are associated with hearing problems such as tinnitus^28^, hyperacusis^29^, and hearing threshold changes^30,31^. Furthermore, there was observed a high-frequency hearing loss in women with PCOS, which is a risk factor for menstrual irregularity^32^. Additionally, female hormone replacement therapy was shown to help alleviate tinnitus in postmenopausal women^33,34^. These previous studies suggest possible associations between sex hormone status and tinnitus in women and consistently support our findings on the association between menstrual cycle irregularity and tinnitus. To the best of our knowledge, our study is meaningful as the first population-based study to identify the association between menstrual cycle irregularity and tinnitus in women of childbearing age. Notably, irregular menstrual cycles with longer durations were more likely to be associated with severe tinnitus.

Our study also found the possible association of mental health problems with menstrual cycle irregularity and tinnitus; several previous studies supported this finding. A greater risk of menstrual cycle irregularity was shown with increased depression and decreased psychological well-being in adult women^17^. In addition, high levels of stress and depressive mood were associated with menstrual cycle irregularity in adolescents and female college students^16,26^. Hormonal changes during the menstrual cycle were associated with suicidal attempts and suicidal ideation in adult women^35^. In particular, irregular menstrual cycles in female adolescents were associated with increased risk for suicidal ideation^36^. Regarding the association between mental health and tinnitus, a study for Egyptian outpatients reported that the duration of exposure to tinnitus was associated with severity of depression, and severity of tinnitus with severity of stress^9^. A study for healthy Koreans also showed that tinnitus occurrence was associated with an increase in perceived stress or stress hormones levels^11^. Moreover, a recent Swedish cross-sectional study reported that severe tinnitus was associated with suicide attempt in women^8^. Although the mechanism linking menstrual cycle irregularity and tinnitus has not been revealed, from collected findings of mentioned previous studies, a possible mediating effect of mental health problems between menstrual cycle irregularity and tinnitus may be inferred. In addition, from evidence that many genetic factors are associated with menstrual cycle^37^ and genetic factor may be predisposed in young people especially for bilateral tinnitus^38^, genetic factors may potentially contribute to the association between menstrual cycle irregularity and tinnitus. Further studies are needed to confirm the associations.

There are some limitations in this study. First, this study is based on retrospective self-reported data; therefore, there may be a recall bias. Second, it is not possible to deduce the causal relationship between menstrual cycle irregularity and tinnitus from cross-sectional design. Third, only questionnaire-based data without objective examinations were used to define menstrual cycle regularity, tinnitus, and mental health problems due to lack of data from the KNHANES, thus it was impossible to identify them more accurately and to reflect fluctuations of them over time. Fourth, menstrual cycle irregularity was classified into three groups based only on longer duration. Although confirmative diagnostic criteria for menstrual cycle irregularity have still not been established, it is necessary to classify menstrual cycle irregularity with more detailed parameters in later studies. Fifth, prevalent mental health problems such as anxiety were not considered due to lack of data from KNHANES. Despite these limitations, the major strength of this study is that it is the first study to confirm the association between menstrual cycle irregularity and tinnitus, suggesting the clinical implications of menstrual cycle irregularity in assessing hearing problems in women. Furthermore, this study used nationally representative data with large samples; thus, it might provide epidemiologic evidence for single ethnicity. Additionally, this study accounted for comprehensive potential confounding factors which might be related to both menstrual irregularity and tinnitus.

In conclusion, our study found the positive associations between menstrual cycle irregularity and tinnitus. Menstrual cycle irregularity may be considered as a related factor in assessing tinnitus in women with child bearing age. Furthermore, our results suggest appropriate interventions to alleviate tinnitus symptoms through accurate assessment and management of menstrual cycle irregularity in women. Further studies such as case-control and cohort studies are warranted to confirm the associations between menstrual irregularity and tinnitus.

## Methods

### Data source and participants

This study used data from the 5th Korea National Health and Nutrition Examination Survey (KNHANES) conducted by the Korea Centers for Disease Control and Prevention (KCDC) for three years from 2010 to 2012. KNHANES has been conducted annually to identify the health and nutritional status of non-institutionalized civilians of South Korea, since 1998. It is designed as a rolling sampling survey method so that each of the three years can be independently representative of the nationwide probability sample. In the KNHANES, a stratified, multi-stage, and clustered probability design was used to improve the accuracy of the representative sample. The survey items include three parts: health interview, health examination, and nutritional surveys. The survey protocol is reviewed and approved by the Research Ethics Review Committee of KCDC. Written informed consent was taken from all participants of the survey. This study followed the principles of the Declaration of Helsinki.

Of the 13,918 female adults who participated in the 5th KNHANES, we excluded those aged <19 or >54 years old (n = 7416), those in menopausal state (n = 1733), those who were pregnant (n = 2), and those with missing data (n* = *134). Finally, data of 4633 female adults were analyzed. The data are accessible at http://knhanes.cdc.go.kr/knhanes/main.do.

### Definition of tinnitus

The presence of tinnitus was assessed based on the participants’ responses to the following question: “Have you experienced tinnitus within the past year (ringing, buzzing, roaring, or hissing sound)?” If participants answered “yes”, they were required to mark one of the following questionnaires responses: (1) My tinnitus is not annoying; (2) My tinnitus is annoying and makes me nervous; (3) My tinnitus is severely annoying and causes sleep problems. Based on the participants’ responses, the severity groups of tinnitus were indicated as mild, moderate, and severe.

### Menstrual cycle characteristics

Menstrual cycle characteristics were assessed by asking the participants to recall their menstrual cycle duration. Participants who responded “yes” to the following question; “Do you have a regular menstrual cycle?” were identified as having regular menstrual cycles, while those who responded “no” were as having irregular menstrual cycles. In addition, if women indicated menstrual cycle irregularity, they were asked about the duration between menstruations (up to 3 months or longer than 3 months).

### Covariates

Participants’ socio-demographic and lifestyle characteristics were assessed through a self-reported questionnaire or by trained interviewers. Educational level was classified into four groups as follows; below elementary school graduated, completed middle school, completed high school, and university graduated or higher. Income level was classified based on the quartile groups of monthly household income. Residential area was divided into urban and rural, and occupation was divided as having a job and unemployed. Smoking status was divided into ex-smokers or current smokers, and non-smokers. Ex-smokers or current smokers were defined as those who are currently smoking or have smoked for at least one day in the past month. Regarding alcohol drinking, individuals who drank more than 30 grams a day were defined as heavy drinkers. Physical activity was assessed by using the International Physical Activity Questionnaire short form for Koreans^39^. Individuals who exercised moderately more than 30 min per session for more than 5 times a week or those who performed vigorous exercise more than 20 min per session for more than three times a week were considered as performing regular physical activity. Sleep duration was assessed by the question “How many hours do you usually sleep a day?” In addition, parity was assessed by the question regarding whether she has had childbirth. Ever use of oral contraceptives was also assessed.

The mental health characteristics were assessed regarding psychological stress, depressive mood, and suicidal ideation. Stress was assessed using the question “How much stress do you usually feel in your daily life?” Participants who responded with a stress level of “severe” or “a lot” were defined as the high-stress group, while those who responded with “a little” or “rare” as the low-stress group. Participants who responded “yes” to the question “Have you felt sad or desperate for two consecutive weeks during the past year?” were defined as having a depressive mood. Participants who responded “yes” to the question “Have you ever considered suicide in the past year?” were defined as having a suicidal ideation.

Trained staff measured participants’ height and weight, and body mass index (BMI) was calculated by the following formula: weight (kg)/height^2^ (m^2^).

### Statistical analysis

The Statistical Analysis System (SAS version 9.3; SAS Institute, Cary, NC, United States) survey procedure was used for statistical analysis. Chi-square test or independent t-test was performed to identify differences in participants’ characteristics according to menstrual cycle irregularity or tinnitus. The results were expressed as percentage (standard error) and mean ± standard error. The odds ratio (OR) and 95% confidence interval (CI) of tinnitus according to menstrual cycle irregularity were calculated using a hierarchical multivariable logistic regression analysis. Model 1 was adjusted for age, smoking status, alcohol drinking, physical activity, parity, and BMI. Model 2 was adjusted for the variables considered in model 2 plus educational level, household income level, sleep duration, ever use of oral contraceptives, psychological stress, and depressive mood.
